# Outcomes of Vitrectomy in Pediatric Retinal Detachment with Proliferative Vitreoretinopathy

**DOI:** 10.1155/2017/8109390

**Published:** 2017-08-03

**Authors:** Robert Rejdak, Dominika Nowakowska, Katarzyna Wrona, Ryszard Maciejewski, Anselm G. Junemann, Katarzyna Nowomiejska

**Affiliations:** ^1^Department of General Ophthalmology, Medical University of Lublin, Lublin, Poland; ^2^Department of Experimental Pharmacology, Medical Research Centre, Polish Academy of Sciences, Warsaw, Poland; ^3^Human Anatomy Department, Medical University of Lublin, Lublin, Poland; ^4^Department of Ophthalmology, University of Rostock, Rostock, Germany

## Abstract

**Aim:**

To report outcomes of pars plana vitrectomy (PPV) in pediatric retinal detachment (RD) with proliferative vitreoretinopathy (PVR), complications, factors influencing the final anatomical and functional results.

**Methods:**

Retrospective consecutive case series of 14 eyes. Average postoperative follow-up period was 34 months.

**Results:**

Mean age of patients was 10 years; eleven patients (79%) were males. The most common etiology was trauma (57%), the second—myopia (36%) and one case of uveitis (7%). At the day of presentation, the best-corrected visual acuity (BCVA) was worse than hand motion (50%); macula was detached in 86% of cases. Simultaneous PPV and phacoemulsification with intraocular lens (IOL) implantation were performed in 12 cases (86%). The most common endotamponade during PPV was silicone oil (93%). Anatomic reattachment was accomplished in 86% of cases. Final BCVA was equal or better than 0.1 in 50% of patients. The postoperative complications were found in 5 eyes (36%).

**Conclusion:**

Complete PPV was allowed for anatomically reattached retina and preserved vision in pediatric complex RD with PVR. However, visual outcomes were not satisfactory. Preserving vision in children with RD is of great importance for their future motor and intellectual development. This trial is registered with ClinicalTrials.gov Identifier: NCT03208205.

## 1. Introduction

The occurrence of retinal detachment (RD) in children is less frequent than that in adults. Pediatric RD constitutes for 3.2–6.6% of all cases of RD [1]. Some publications indicate that the frequency of RD in children is higher and may affect even 12.6% of all patients suffering from RD [2]. There are many differences between pediatric and adolescent RDs. RD in children differs in the etiology, anatomical characteristics, and prognosis [3, 4]. Ocular comorbidities, such as congenital developmental anomalies, myopia and trauma, or previous intraocular surgeries, have been reported to be the most prevalent predisposing factors for pediatric RD [2, 4]. As reported in previous studies, anatomical success of RD surgery in pediatric patients is lower than that in adults—from 10% to 80% with different surgical approaches [5, 6]. Consequently, pediatric RD is a great challenge for ophthalmologists.

Children usually present late and have clinical features of longstanding RD such as macular involvement and proliferative vitreoretinopathy (PVR). Histologically, PVR is the formation of abnormal cellular accumulations in the vitreous cavity and subretinal space produced by the retinal pigment epithelium (RPE) and Müller cells [7]. The PVR process is characterized by formation of periretinal fibrocellular membranes, intraretinal fibrosis, and subretinal bands [8]. Treatment of RD in eyes with PVR is challenging and requires complex vitreoretinal surgery.

The aim of this study was to review outcomes of pars plana vitrectomy (PPV) in pediatric RD accompanied with PVR as well as factors influencing the final anatomical and functional results.

## 2. Methods

This is the retrospective study of 14 consecutive patients younger than 18 years of age who underwent primary PPV at the Department of General Ophthalmology of Medical University of Lublin in a time period from 1st January 2006 to 1st January 2017. This study followed the tenets of the Declaration of Helsinki. The treatment chosen in the study was a part of a standard care. Written informed consent was taken from all subjects. All patients underwent vitreoretinal surgery due to RD (rhegmatogenous, tractional, or combined rhegmatogenous and tractional). Exclusion criteria from the study was the time of follow-up less than 6 months.

The average age at the time of the presence of RD was 10 ± 4.7 years (range 4–17 years). Pre- and postoperative data were collected. Descriptive statistical analysis included gender, age at the presentation, laterality, etiology, duration of presenting symptoms, presences of ocular comorbidities, macular status (attached or nonattached), presence of PVR grade C, initial and final best-corrected visual acuity (BCVA), number of procedures, type of endotamponade during PPV, final lens status, duration of the follow-up, anatomical success, and complications. Indications for PPV were as follows: presence of advanced PVR and/or total RD and/or multiple breaks, giant retinal tears. PVR was graded according to the Retina Society Terminology Classification [9]. Visual acuity was measured by Snellen charts. The anatomical success was defined as persistent retinal reattachment at the last follow-up visit (regardless of presence or absence of silicone oil tamponade).

Statistical computations were performed using STATISTICA 13PL (Stasoft, USA) programme.

## 3. Surgical Procedure

Surgeries were performed under general anesthesia. Complete 23G PPV with scleral indentation was performed using the Constellation system (Alcon, Fort Worth, Texas, US). Posterior vitreous detachment (PVD) was induced very carefully by the cutter probe using aspiration. An amount of fifty microliters of triamcinolone acetonide (TA) aqueous suspension (Kenacort 40 mg/ml, Bristol-Meyers Squibb, Princeton, NJ, USA) was injected into the midvitreous with a cannula and entrapped in the vitreous gel. Additional delamination of posterior hyaloid was required in half of the cases. At this step, high magnification was achieved by the macula lens (60D) of binocular indirect ophthalmo-microscope (BIOM). The posterior hyaloid was undermined with 25G needle and carefully dissected by PPV probe. No vitreous was left attached to the retina posterior to the vitreous base. There were no iatrogenic breaks created during PVD induction. Peeling of the epiretinal membranes (ERMs) and internal limiting membrane (ILM) was performed after staining with Brilliant Blue G. A heavy perfluorocarbon liquid (perfluoronoctane, HPFCL) was injected slowly up to the posterior side of the retinal break to stabilize the central retina in all eyes. The peripheral vitreous was removed in all eyes with 360° of the vitreous base shaved under scleral indentation. Subretinal membranes were extracted wherever they caused significant traction. If retinal attachment was not achieved, relaxing retinectomy was performed after endodiathermy. After complete retinal attachment was achieved, endolaser photocoagulation was applied around the retinal break(s) as three to four rows of burns or/and cryopexy under HPFCL. Then, a HPFCL/air/5000cst silicone oil exchange was performed. In one eye, only HPFCL/air exchange was performed. Sclerotomies were sutured when needed with Vicryl 8.0 sutures.

## 4. Results

Clinical features of pediatric RD at the day of presentation and further status of the retina are detailed in Table 1.

Eleven patients (79%) were males. The right eye was equally involved as well as the left eye (50%). Visual acuity was improved after surgery in 50% (*n* = 7) and was the same in 5 patients (36%). Trauma was the most prevalent (57%, *n* = 8) cause of RD, while the second one was high myopia (spherical equivalent ≤ −6.0 D) 36% (*n* = 5); in one case (7%), uveitis was diagnosed. Duration of presenting symptoms reported by patients and their parents oscillated around 8.57 ± 16 days. Demographical and clinical data of each patient in the study group are presented in Table 2. Preoperative BCVA was as follows: light perception in 6 patients (42.8%), hand motion in 1 patient (7.1%), counting fingers in 1 patient (7.1%), 0.004–<0.1 in 4 patients (28.6%), 0.1 in 2 patients (14.3%), and 0.2 in 2 patients (14%). Final BCVA was as follows: light perception in 3 patients (21.4%), hand motion in 1 patient (7.1%), counting fingers in 2 patients (14.3%), 0.001–0.004 in 4 patients (28.6%), 0.5 in 1 patient (7.1%), and 0.6 in 1 patient (7.1%).

Most cases were pure rhegmatogenous RDs (79%). In all cases, PVR grade C was reported. Most of the eyes presented detached macula at the time of diagnosis (86%). In 12 eyes (86%), simultaneous phacoemulsification with intraocular lens (IOL) implantation was performed during the first surgery. In one case (7%), cataract surgery was performed during the second approach, and in another one case, the eye remained phakic after silicone oil removal. The most common endotamponade was silicone oil (93%). In one case, because of the massive vitreous hemorrhage and peripheral RD, air was used as a tamponade. The average number of surgeries was 2.3 ± 1.14. In 4 patients (29%), two surgical procedures were performed. Twenty-six percent of subjects underwent only one procedure. In one case (7%), the number of surgeries was five. Anatomical success was achieved in 86% of cases. Silicone oil was finally removed in 6 cases. Average time of the presence of silicone oil in the vitreous cavity was 10 months in these eyes. After the last surgery (silicone oil removal), patients were kept under strict control of the mean of 31 months. Moreover, in this patient group, we did not observe retinal redetachment. In another 4 cases, silicone oil was exchanged and 3 eyes remained silicone oil-sustained, and in one case, air was used as a tamponade and the retina remained attached at the end of the follow-up. Different postoperative complications, such as silicone oil under conjunctiva, posterior capsule opacity, temporary ocular hypertension, iris and corneal neovascularisation, band keratopathy, postoperative mydriasis, and retinal redetachment, were observed in 5 patients (36%).

## 5. Discussion

The current study was performed to delineate the clinical characteristics and surgical outcomes of pediatric RD accompanied with PVR. Predisposing factors or underlying retinal conditions were present in 100% of the analyzed patients; trauma was the most important etiologic factor (57.1%) followed by high myopia (35.7%). As reported in previous studies, trauma is the most often cause of pediatric RD, ranging from 36 to 45% [10, 11]. Soheilian et al. also evaluated the clinical features and functional and anatomical outcomes after surgical intervention in pediatric rhegmatogenous RD and found that trauma and congenital developmental anomalies are leading etiologies in pediatric rhegmatogenous RD [12]. In many studies, myopia is indicated to be a common etiological factor of pediatric RD (18.1–38%) [2, 13, 14]. We also found a strong male predominance (79%) similar to another series [13]. The higher rate of pediatric RD in males may be due to higher exposure to trauma due to gender pattern of behavior. In our study, RD was diagnosed at the mean age 10 which is consistent with other studies [2, 4]. In this age range, trauma and congenital or developmental anomalies were the most common etiologies. We concluded that the higher rate of RD occurrence at the age of 10 may be related to more susceptibility to trauma as well as the progressive nature of congenital and structural pathologic processes. In one of the studies, it has been reported that bilateral RD is more common in children [4]. In the current study, about 80% of RDs were rhegmatogenous. All of the patients required PPV as the initial surgical procedure which was due to the complexity of RD and presence of PVR, giant tears, and multiple retinal breaks. Silicone oil was necessary for intraocular tamponade in most of the patients (93%). Final anatomical success rate was 86% in our study. Our anatomical success rates were similar to the previous studies. Butler et al. analyzed 15 cases and reported similar success rates (86.6%) [5]. Gurler et al. reported attached retina in 80% children after primary surgery [15]. They also indicate significantly higher final BCVAs in children with trauma-associated RD than in group with myopia-associated RD. We have not found a similar relationship. The authors explain better functional outcomes after surgery in RD caused by trauma, immediate admission to the hospital without a delay after an ocular trauma.

Interestingly, in the current study, retina remained detached only in two cases of total posttraumatic RD.

In pediatric subjects, PPV seems to be more difficult and challenging due to the strong vitreororetinal adhesions and few areas with posterior vitreous detachment. Therefore, an external approach such as scleral buckling (SB) is chosen by some surgeons [2]. Errera et al. in their retrospective consecutive case series reported outcomes of primary SB procedures for pediatric RD. They achieved retinal reattachment with 1 operation in 73% of cases (76 of 104 eyes). The authors found the factors associated with a statistically significant increased risk of failure. Based on their results, it can be concluded that primary SB is a proper solution in pediatric RD excluding cases with more than one break, three or more quadrants of detachment, horseshoe tears, no breaks seen on preoperative examination, and Stickler syndrome [16].

In our study, we have found that 93% PPV was combined with phacoemulsification and IOL implantation. Therefore, we deliberated that phacoemulsification with IOL implantation allows for more proper management of the vitreous base. Near-complete removal of the vitreous and all tractional membranes is important in primary surgery for good retinal reattachment rate. Although there have been various advances in PPV techniques and equipment, the transparency of the vitreous can pose some difficulties during PPV. Peyman et al. first described the use of intravitreal TA as an aid to visualize the vitreous and the posterior hyaloid during PPV [17]. We used TA intraoperatively to remove strongly attached vitreous efficiently. Intraoperative use of TA has been already reported to increase the visibility of the hyaloid and ERM, allowing more complete and safer PVD [18, 19]. Enaida et al. studied the advantages of TA-assisted PPV for various retinal diseases and reported that it may reduce the incidence of reoperation due to preretinal fibrosis [20]. TA reduces also postoperative inflammatory response which is very high in children [21]. Bimanual technique for dissection of the posterior hyaloid is very well established in diabetic RD [22]. PPV combined with delamination and dissection of the preretinal fibrovascular membranes provides relief of the retinal traction and is one of the major causes of the anatomical success.

Final BCVA in our study was unsatisfactory which may be due to the high incidence of trauma as the cause of RD in our series. The morphologic and functional outcomes of traumatic RD surgery are not favorable due to the longer duration of RD, frequent macular involvement, and high rate of PVR [13]. The longest period of time of presenting symptoms in currently reviewed group was 60 days. The duration time was determined by reported symptoms. Children, especially in lower age, do not notice changes in visual field or acuity. Even if they found disturbance in vision, they postpone the compliance because of the fear associated with the hospitalization, examination, and treatment. Late diagnosis in pediatric patients leads to more frequent macula-off status, with extensive multiple-quadrant RD and higher rates of PVR development. In our series, macula-off status was detected in 86% at presentation, similar to 80% [2] and 81.9% [14] reported by other investigators. On the contrary, in another study, lower rate (50%) of macula-off status was reported but still it was due to late diagnosis, as described by the authors [10]. In our research, PVR Grade C was noted in 100% of eyes at presentation and was higher than in other studies (70% [10] and 69.1% [14]). Higher rate of advanced PVR is probably associated to late diagnosis and in most cases posttraumatic changes in eyeball. Moreover, in the pediatric age group, delayed diagnosis accompanied with a higher degree of intraocular cellular activity and proliferation may result in higher PVR occurrence.

The mismatch between anatomical and functional success rates may be due to the high risk of amblyopia in children; however, other factors, such as corneal irregularity due to corneal laceration, simultaneous traumatic optic neuropathy or choroidal detachment, and the presence of PVR, may also be important in functional visual loss.

The current case series study is limited by its retrospective nature and relatively small group of patients.

## 6. Conclusions

In summary, pediatric RD is commonly associated with an underlying condition. Although children often present chronic RD and PVR, the anatomical outcomes following surgical intervention are favorable in most cases. A higher number of RD procedure in children, compared to adults, are associated with worse anatomical outcomes. Anatomic and functional outcomes in pediatric RD are not as good as those in adults. Preserving vision in children with RD is of great importance for their future motor and intellectual development.

## Figures and Tables

**Table 1:** Characteristics of retinal detachment (RD) in a presented study group of 14 children.

Parameter		Number of subjects	Percentage of subjects
Etiology	High myopia	5	35.7
	Trauma	8	57.1
	Uveitis	1	7.1

Type of retinal detachment	Mixed rhegmatogenous and tractional detachment	2	14.3
	Rhegmatogenous detachment	11	78.6
	Tractional detachment	1	7.1

Status of the macula	On	2	14.3
	Off	12	85.7

Anatomical success	Persistent retinal reattachment	12	85.7
	Retina detached	2	14.3

Endotamponade	Silicone oil	13	92.3
	Air	1	7.14

**Table 2:** Patients' data (age, gender, and initial and final best-corrected visual acuity—BCVA).

Patient no.	Age (years)	Gender (F: female, M: male)	Initial BCVA^∗^ (Snellen chart)	Final BCVA (Snellen chart)	Duration of the follow-up (months)	Surgical management (number of operations)	Postoperative complications	Average time to remove silicone oil (months)
1	9	F	0.04	0.06	42	(1) phaco^∗∗^ + IOL^∗∗∗^ + PPV + ILM peeling^∗∗∗∗^ + +silicone oil (2) PPV + silicone oil exchange (3) silicone oil removal		12

2	12	M	Counting fingers	Counting fingers	38	(1) phaco + IOL + PPV++ air		

3	15	M	0.01	0.1	44	(1) phaco + IOL + PPV + silicone oil (2) silicone oil exchange (3) silicone oil removal	Silicone oil under conjunctiva	14

4	10	M	Light perception	0.004	29	(1) phaco + IOL + PPV + ILM peeling + silicone oil	Posterior capsule opacity	

5	17	M	0.04	0.2	6	(1) phaco + IOL + PPV + silicone oil		

6	16	M	0.04	0.04	49	(1) phaco + IOL + PPV + ILM peeling + silicone oil (2) silicone oil removal		13

7	17	F	Light perception	0.1	29	(1) Phaco + IOL + PPV + silicone oil (2) silicone oil removal + SF6^∗∗∗∗∗^ gas		5

8	4	F	Hand motion	Counting fingers	32	(1) PPV + silicone oil (2) silicone oil removal		8

9	5	M	Light perception	Light perception	41	(1) phaco + IOL + PPV + silicone oil (2) silicone oil exchange	Iris and corneal neovascularisation, retinal redetachment	

10	4	M	0.5	0.1	11	(1) phaco + IOL + PPV + silicone oil		

11	9	M	Light perception	Hand motion	74	(1) PPV + silicone oil (2) phaco + IOL + PPV + silicone oil exchange (3) PPV + silicone oil removal + SF6 gas (4) PPV + silicone oil (5) PPV + silicone oil exchange	Temporary ocular hypertension	

12	6	M	Light perception	Light perception	36	(1) phaco + IOL + PPV + silicone oil (2) PPV + silicone oil exchange (3) chelation + PPV + silicone oil exchange	Band keratopathy and postoperative mydriasis, retinal redetachment	

13	8	M	Light perception	Light perception	20	(1) phaco + IOL + PPV + ILM peeling +silicone oil (2) silicone oil exchange (3) silicone oil exchange		

14	8	M	0.6	0.2	20	(1) phaco + IOL + PPV + silicone oil (2) silicone oil exchange (3) silicone oil removal		8

^∗^BCVA: best-corrected visual acuity. ^∗∗^Phaco: phacoemulsification. ^∗∗∗^IOL: intraocular lens. ^∗∗∗∗^ILM: internal limiting membrane. ^∗∗∗∗∗^SF 6: sulphahexafluoride.
